# Identification of Metabolic Pathways Expressed by *Pichia anomala* Kh6 in the Presence of the Pathogen *Botrytis cinerea* on Apple: New Possible Targets for Biocontrol Improvement

**DOI:** 10.1371/journal.pone.0091434

**Published:** 2014-03-10

**Authors:** Anthony Kwasiborski, Mohammed Bajji, Jenny Renaut, Pierre Delaplace, M. Haissam Jijakli

**Affiliations:** 1 Plant Pathology Unit, Gembloux Agro-Bio Tech/University of Liège, Gembloux, Belgium; 2 Proteomics Platform, Department of Environment and Agrobiotechnologies/Centre de Recherche Public Gabriel Lippmann, Belvaux, Luxemburg; 3 Plant Biology Unit, Gembloux Agro-Bio Tech/University of Liège, Gembloux, Belgium; Nanjing Agricultural University, China

## Abstract

Yeast *Pichia anomala* strain Kh6 Kurtzman *(Saccharomycetales: Endomycetaceae*) exhibits biological control properties that provide an alternative to the chemical fungicides currently used by fruit or vegetable producers against main post-harvest pathogens, such as *Botrytis cinerea (Helotiales: Sclerotiniaceae)*. Using an *in situ* model that takes into account interactions between organisms and a proteomic approach, we aimed to identify *P. anomala* metabolic pathways influenced by the presence of *B. cinerea*. A total of 105 and 60 *P. anomala* proteins were differentially represented in the exponential and stationary growth phases, respectively. In the exponential phase and in the presence of *B. cinerea*, the pentose phosphate pathway seems to be enhanced and would provide *P. anomala* with the needed nucleic acids and energy for the wound colonisation. In the stationary phase, *P. anomala* would use alcoholic fermentation both in the absence and presence of the pathogen. These results would suggest that the competitive colonisation of apple wounds could be implicated in the mode of action of *P. anomala* against *B. cinerea*.

## Introduction

Post-harvest diseases cause significant economic losses for fruit and vegetable producers. From 20–25% of the production in industrialised countries and up to 35–50% in developing countries [1], [2] are lost. The control of post-harvest diseases is mainly based on the use of chemical fungicides [3]. With the increasing consumer demand for chemical residue-free products and the emergence of resistant fungi, the interest of the producers in alternative solutions has increased. One of the most promising technologies is the use of antagonistic organisms. Some antagonist-based products are already commercially available and others are currently at varying stages of development [3], [4]. Understanding the mode(s) of action of antagonists is one of the parameters of product development and is relevant for marketing purposes [5]. It allows for the identification of useful traits that could be upgraded by genetic tools and improves performance reliability through the development of formulations [6]. Competition for space and nutrients [7]–[12], secretion of lysis enzymes [6], [13], [14] and activation of plant defences [15] have already been reported to play a role in the antagonistic mode(s) of action. However, the cellular mechanisms that support these modes of action are not clear. Recently, cDNA-AFLP was used on an *in situ* model close to the one used in this study, to search for *Candida oleophila* strain O genes of biocontrol relevance against *B. cinerea* on apples [16] and identified primary metabolisms that could be involved in the colonisation of apple wounds.

*Pichia anomala* strain Kh6 (*Saccharomycetales: Endomycetaceae*) was identified as an antagonist of the apple pathogen *Botrytis cinerea (Helotiales: Sclerotiniaceae)* [17]. Microbiological, biochemical and molecular approaches [13], [18]–[21] revealed the complexity of *P. anomala* mode of action. Jijakli and co-workers [17] demonstrated that there was a quantitative relationship between pathogen spore concentration and the amount of the antagonist needed for disease control on apple, suggesting that the protective effect of the antagonistic yeast was closely related to wound colonisation [17], [22]. When cultured on a medium supplemented with *B. cinerea* cell walls as the sole carbon source, *P. anomala* overexpressed genes that exhibited β-glucosidase, transmembrane transport, citrate synthase and external amino acid sensing and transport functions [20]. More particularly, in these conditions, a higher activity of β-glucanases (PAEXG1 and PAEXG2) was detected [13]. When mutant strains of *P. anomala* strain K (with two genes coding for PAEXG1 and PAEXG2 disrupted separately or simultaneously) were applied on apple wounds, their biocontrol efficiency against *B. cinerea* decreased [21]. However, the study also highlighted that both high yeast concentrations and apple ripening stage appeared to compensate for the inactivation of the PAEXG genes [21]. Although the above-mentioned studies identified different modes of action of *P. anomala* against *B. cinerea*, however, cellular mechanisms implied in this biocontrol still remain to be identified.

Proteomic techniques couple bi-dimensional electrophoresis (2-DE) with mass spectrometry to separate and identify hundreds of proteins present in a complex mixture and is powerful to observe changes in protein expression in organisms under various environmental conditions [23]. Regarding biocontrol, proteomics is a promising technique for identifying the mechanisms associated with the biocontrol process [24]–[26] and for optimising formulations and enhancing antagonist efficacy.

The purpose of this work was to compare the proteome of *P. anomala* grown on apple fruit in the absence or presence of *B. cinerea* and at different growth phases in order to identify *P. anomala* influenced metabolic pathways while taking into account the antagonist/pathogen/host tripartite interaction as in natural infection conditions. Cellular processes potentially involved in the protective effect of *P. anomala* are also proposed.

## Materials and Methods

### Organisms and model

All the experiments were carried out *in situ* on *Malus x domestica* cv. Golden Delicious purchased from a local supplier (Van Dyck Freres SA, Namur, Belgium). To study the yeast proteome within the triple apple/antagonist/pathogen interaction, an *in situ* model was recently developed [27] that allows for nutrient exchanges between apples and microorganisms and for the extraction of proteins compatible with a 2-DE study, both quantitatively and qualitatively. Forty and ten apples were used in the exponential (7 hrs) and stationary (24 hrs) growth phases, respectively. Four different samples corresponding to *P. anomala* incubated alone for 7 hrs (Kh6-1) or for 24 hrs (Kh6-2) and *P. anomala* co-inoculated with *B. cinerea* for 7 hrs (Kh6B-1) or for 24 hrs (Kh6B-2) were produced in five biological replicates.

### Protein extraction, two-dimensional electrophoresis, image analysis and protein identification by mass spectrometry

The hot SDS/acetone protein extraction protocol and 24 cm 2-DE procedure based on Delaplace et al. (2006) [28] were adapted and are detailed by Kwasiborski et al. [27]. The protein content was determined using the Bradford method. The obtained 2-DE gels were scanned using the Typhoon Variable Mode Imager 9400 (GE Healtcare) at a resolution of 100 µm and analysed using 2-DE image analysis software Decyder v7 (GE Healthcare). The proteome of *P. anomala* inoculated alone on apple wounds was compared to the proteome of *P. anomala* in the presence of *B. cinerea* in the exponential and stationary growth phases: Kh6-1 *vs.* Kh6B-1 after 7hrs and Kh6-2 *vs.* Kh6B-2 after 24hrs.

Statistical procedures for the selection of spots of interest were carried out using XLstat addinsoft version 7.5.3 (Microsoft Corporation, Seattle, USA). The normal distribution of our data was verified using the Shapiro-Wilcoxon test and proteins with abnormal distributions were normalised using the Box-Cox procedure. Protein abundances were considered as statistically different between our conditions using a one way analysis of variance (p<0.05). Then differentially expressed proteins across all biological replicates with an absolute ratio of at least 1.5-fold were selected.

The mass spectrometry identifications were carried out by the proteomic platform based in the Centre de Recherche Public G. Lippmann (Belvaux, Luxembourg). Spots of interest were excised using the Ettan Spot Picker from the Ettan Spot Handling Workstation (GE Healthcare). After washing and desalting in 50 mM ammonium bicarbonate/50% v/v methanol, followed by 75% v/v ACN, spots were then digested with 10ng/µL Trypsin Gold (MSgrade, Promega, Madison, WI, USA) in 20 mM ammonium bicarbonate. The peptides were extracted using a 50% ACN containing 0.1% TFA and analysed using the Applied Biosystems 4800 Proteomics Analyser (Applied Biosystems, Foster City, CA, USA). Calibration was carried out with the peptide mass calibration kit for 4700 (Applied Biosystems). Proteins were identified from their peptide mass fingerprintings and searching the NCBI protein sequence database using the MASCOT software (Matrix Science, http://www.matrixscience.com, London, UK). The search parameters allowed for carboxyamidomethylation of cysteine as fixed modification and oxidation of methionine, oxidation, dioxydation and kynurenin of tryptophan as variable modifications. After manual checking of the identifications, proteins with at least two matching peptides and a total ion score above 60 were considered as significantly identified.

## Results

**Figure 1:**
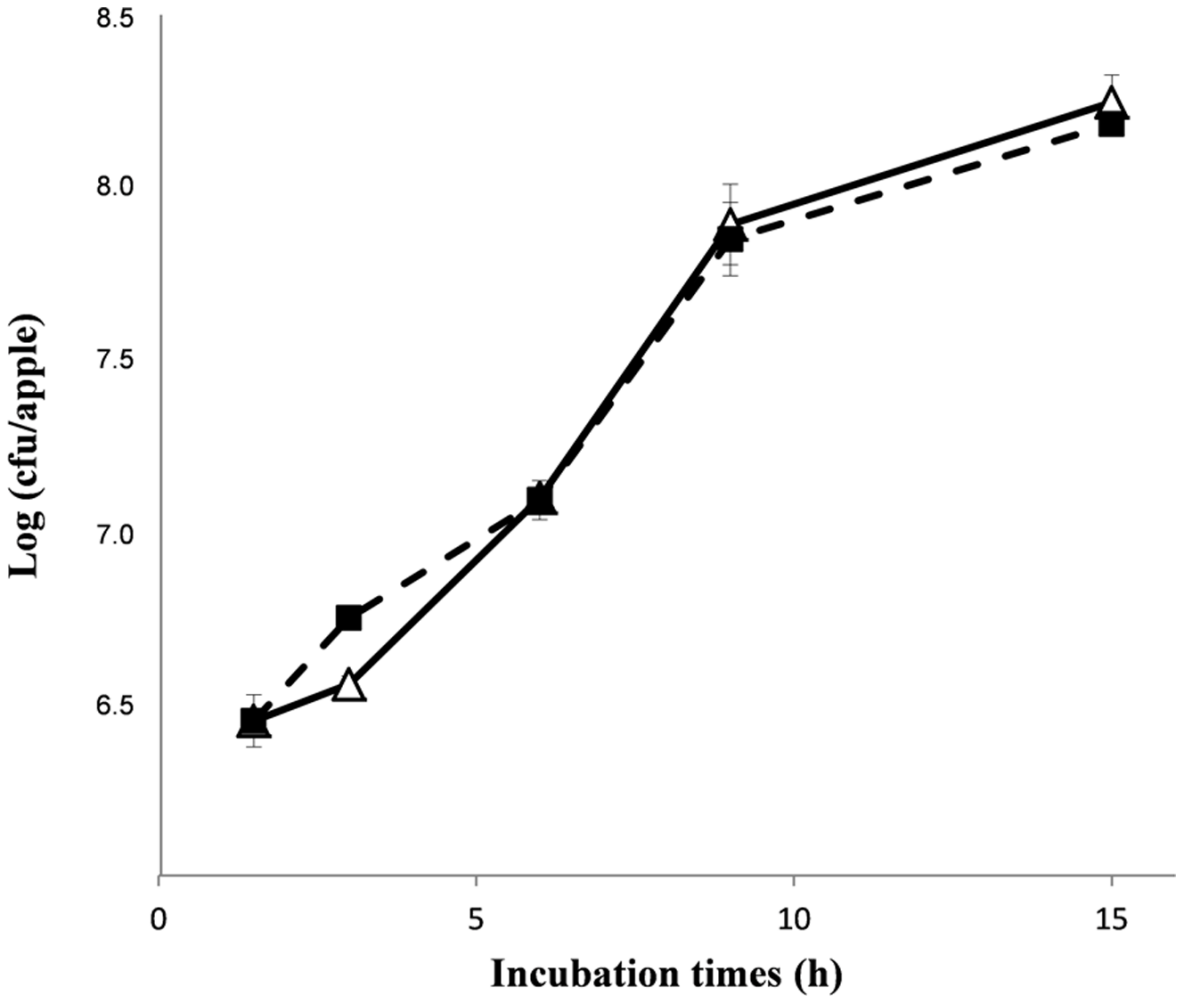
Growth time-courses of *Pichia anomala* Kh6 in the presence (triangle) or absence (filled square) of *Botrytis cinerea* using the apple model. Vertical bars indicate the standard deviation of the log (cfu/apple) determined as a mean of 3 measurements.

Figure 1 presents the growth time-course of *P. anomala* Kh6 in the presence or absence of *B. cinerea* on the developed apple model. *P. anomala* Kh6 grew in the same way in the absence and presence of *B. cinerea* and showed the usual phases, i.e. latent (0 to 2 hrs), exponential (2 to 10 hrs) and stationary (from 10 hrs onward).

One hundred and five protein spots in the exponential growth phase and 60 spots in the stationary growth phase displayed significant differences depending on the absence or presence of *B. cinerea*. Out of these, 61% and 38% were successfully identified by mass spectrometry in the exponential and stationary phases, respectively (Table 1). The remaining proteins were either not found in the databases or quantities in the excised spots were too low to allow for identification by mass spectrometry.

**Table 1:** Metabolism types and metabolic pathways of the *P. anomala* proteins influenced by the absence (Kh6-1 and Kh6-2) or the presence (Kh6B-1 and Kh6B-2) of *B. cinerea* in the exponential (Kh6-1 and Kh6B-1) and stationary (Kh6-2 and Kh6B-2) phases according to the KEGG database (http://www.genome.jp/kegg/kegg2.html).

Metabolism	Metabolic pathway	Number of proteins over-represented in			
		Kh6-1	Kh6B-1	Kh6-2	Kh6B-2
Genome expression	Nucleotide metabolism	1	3	0	2
	Transcription	0	2	0	0
	Amino acid metabolism	5	4	0	2
	Translation	7	8	0	2
Carbohydrate metabolism	Glycolysis	3	5	3	5
	Citric acid cycle	1	1	0	1
	Pentose phosphate pathway	0	5	0	0
	Alcoholic fermentation	0	0	3	2
	Miscellaneous	1	1	0	0
Energetic metabolism	Oxidative phosphorylation	1	7	1	0
Cellular metabolism	Protein folding	1	5	0	0
	Regulation	0	2	2	0
Cell process	Replication	0	1	0	0
Unidentified		22	19	11	26
Total identified		20	44	9	14
Total		42	63	20	40

### Proteome changes during the exponential phase

Table 2 shows the *P. anomala* proteins up- or down-regulated by the absence or presence of the pathogen during the exponential phase. The abundance of forty four proteins was decreased, while the abundance of 63 was increased in the presence of *B. cinerea*. These proteins are implied in a broad range of metabolic pathways: genome expression, carbohydrate, energy and cellular metabolisms and cell process (Table 1).

**Table 2:** Identification of proteins exhibiting different levels of expression according to the absence (Kh6-1) or the presence (Kh6B-1) of *B. cinerea* in the exponential phase.

Spot no.	Protein name^a^	Accession no.^a^	Organism	Mascot score^b^	Theoretical pI/MW^c^	Spot quantity (ppm)^d^		p^e^
						Kh6-1	Kh6B-1	
Genome expression								
***Nucleotide metabolism***								
378	Adenylosuccinate synthase	gi|149247068	*Lodderomyces elongisporus*	77	6.77/48.0	2.36	**4.99**	*
399	Adenylosuccinate synthase	gi|149247068	*Pichia tropicalis*	293	6.77/48.0	0.00	**3.26**	***
492	Glutamine synthetase	gi|254574242	*Pichia pastoris*	178	6.29/41.8	0.00	**6.92**	***
944	Guanylate kinase	gi|126137291	*Pichia stipitis*	120	5.36/21.6	**9.96**	3.99	*
***Transcription***								
354	ATP-dependent RNA helicase sub2	gi|2500534	*Sclerotinia sclerotiorum*	112	5.29/50.3	0.00	**3.28**	***
368	ATP-dependent RNA helicase sub2	gi|2500534	*Sclerotinia sclerotiorum*	152	5.29/50.3	9.06	**27.08**	***
***Amino acid metabolism***								
79	Methionine synthase	gi|47132400	*Saccharomyces cerevisiae*	116	5.84/86.0	**9.46**	3.34	***
80	Methionine synthase	gi|47132400	*Pichia pastoris*	86	5.84/86.0	**17.44**	10.95	**
238	Thiamine pyrophosphate enzyme	gi|310796355	*Glomerella graminicola*	850	5.96/63.3	0.00	**1.82**	***
336	NADP-specific glutamate dehydrogenase	gi|190407665	*Saccharomyces cerevisiae*	637	5.56/49.6	0.00	**18.58**	***
352	Homocitrate synthase	gi|149238586	*Scheffetsomyces stipitis*	335	7.25/47.1	**0.88**	0.52	*
418	S-adenosyl-L-homocysteine hydrolase	gi|254572033	*Pichia pastoris*	185	6.20/49.1	0.80	**3.85**	***
539	Acetohydroxy-acid isomeroreductase	gi|957238	*Saccharomyces cerevisiae*	156	9.06/44.4	**9.32**	3.45	**
1048	NADP-specific glutamate dehydrogenase	gi|190407665	*Saccharomyces cerevisiae*	637	5.56/49.6	**13.93**	2.65	***
1421	Acetohydroxy-acid isomeroreductase	gi|957238	*Saccharomyces cerevisiae*	436	9.06/44.4	5.51	**22.22**	***
***Translation***								
131	Translation elongation factor 2	gi|6320593	*Saccharomyces cerevisiae*	105	5.92/93.3	**6.80**	2.82	***
135	Glycyl-tRNA synthetase	gi|302410789	*Verticillium albo-atrum*	992	5.59/74.1	0.00	**2.74**	***
199	Heat shock protein SSB1	gi|149238586	*Lodderomyces elongisporuc*	511	5.18/66.6	**12.82**	7.17	**
207	Heat shock protein SSB1	gi|149238586	*Lodderomyces elongisporuc*	350	5.18/66.6	**5.60**	2.19	**
213	Heat shock protein SSB1	gi|149238586	*Lodderomyces elongisporuc*	72	5.18/66.6	**1.09**	0.32	**
222	Heat shock protein SSB1	gi|149238586	*Lodderomyces elongisporuc*	103	5.18/66.6	0.24	**2.14**	***
225	Heat shock protein SSB1	gi|149238586	*Lodderomyces elongisporuc*	197	5.18/66.6	0.00	**4.57**	***
300	Heat shock protein SSB1	gi|149238586	*Lodderomyces elongisporuc*	89	5.18/66.6	1.80	**3.18**	*
451	ATP-dependent RNA helicase eIF4A	gi|154324134	*Schizosaccharomyces pombe*	296	4.85/44.7	1.68	**3.60**	*
469	Mitochondrial GTPase elongation factor Tu	gi|91178575	*Pichia anomala*	262	6.64/48.0	7.58	3.88	***
680	Elongation factor 1β	gi|156053087	*Sclerotinia sclerotiorum*	212	4.13/22.6	2.98	**6.32**	*
685	Heat shock protein SSB1	gi|149238586	*Lodderomyces elongisporuc*	99	5.18/66.6	0.00	**3.52**	***
696	40S ribosomal protein S0	gi|119490951	*Neosartorya fischeri*	240	4.47/28.0	**36.34**	22.41	***
1433	Translation elongation factor 2	gi|6320593	*Saccharomyces cerevisiae*	106	5.92/93.3	4.66	**7.32**	*
1460	40S ribosomal protein S0	gi|119490951	*Neosartorya fischeri*	154	4.47/28.0	**1.64**	0.25	*
**Carbohydrate metabolism**								
***Glycolysis***								
333	2,3-bisphosphoglycerate-independent phosphoglycerate mutase	gi|310791993	*Glomerella graminicola*	827	5.52/57.5	**1.07**	0.48	**
441	Enolase 1	gi|238850305	*Clavispora lusitaniae*	277	5.55/47.1	**19.05**	9.18	***
452	Enolase 1	gi|238850305	*Clavispora lusitaniae*	317	5.55/47.1	**8.05**	2.33	**
594	Enolase 1	gi|238850305	*Clavispora lusitaniae*	92	5.55/47.1	3.17	**4.98**	***
623	Pyruvate dehydrogenase beta subunit	gi|320582536	*Pichia angusta*	261	5.10/39.3	0.00	**4.02**	***
683	Enolase 1	gi|238850305	*Clavispora lusitaniae*	150	5.55/47.1	0.00	**2.71**	***
686	Fructose 1,6-biphosphate aldolase	gi|320580353	*Pichia angusta*	75	5.90/39.3	1.01	**3.71**	**
814	Triosephosphate isomerase	gi|156040910	*Sclerotinia sclerotiorum*	476	5.52/27.0	1.50	**7.17**	***
***Citric acid cycle***								
187	Succinate dehydrogenase cytochrome b	gi|151941666	*Saccharomyces cerevisiae*	1186	10.6/22.3	1.02	**2.65**	***
506	Succinyl-CoA synthetase beta subunit	gi|119410507	*Neosartorya fischeri*	116	5.62/47.8	**7.09**	4.47	*
***Pentose phosphate pathway***								
120	Transketolase	gi|320589066	*Grosmannia clavigera*	981	6.01/74.7	0.00	**8.69**	***
121	Transketolase	gi|320589066	*Grosmannia clavigera*	981	6.01/74.7	1.51	**4.69**	**
351	6-phosphogluconate dehydrogenase	gi|577839	*Saccharomyces cerevisiae*	353	6.19/53.5	0.00	**18.66**	***
390	6-phosphogluconate dehydrogenase	gi|577839	*Saccharomyces cerevisiae*	137	6.19/53.5	3.72	**4.44**	***
631	Transaldolase	gi|322697295	*Metarhizium acridum*	409	6.49/35.4	4.49	**17.60**	***
***Miscellaneous***								
498	Mannose-1-phosphate guanyltransferase	gi|894204	*Saccharomyces cerevisiae*	159	5.95/39.5	**8.85**	5.34	*
724	L-xylulose reductase	gi|320584091	*Pichia angusta*	134	5.74/29.0	0.00	**4.32**	***
**Energetic metabolism**								
***Oxidative phosphorylation***								
194	Likely vacuolar ATPase V1 complex subunit a	gi|68468638	*Candida albicans*	137	5.23/63.3	2.34	**17.44**	***
375	Cytochrome Bc1 complex subunit 1, mitochondrial	gi|136693	*Saccharomyces cerevisiae*	326	6.78/50.2	1.38	**3.84**	***
382	ATP synthase beta chain, mitochondrial	gi|156050413	*Sclerotinia sclerotiorum*	242	5.24/55.7	1.93	**6.31**	**
385	F1F0-ATPase complex, F1 beta subunit	gi|126134912	*Pichia stipitis*	544	5.28/53.6	**12.10**	6.93	**
609	Inorganic pyrophosphatase	gi|156058103	*Sclerotinia sclerotiorum*	219	5.51/32.2	0.00	**7.64**	***
736	Mitochondrial F-ATPase beta subunit	gi|91178130	*Pichia anomala*	110	4.78/36.2	0.00	**3.90**	***
1412	Cytochrome c oxidase subunit VI	gi|171295	*Saccharomyces cerevisiae*	170	5.80/17.3	0.00	**0.22**	***
1444	Subunit b of the stator stalk of mitochondrial F1F0 ATP synthase	gi|320580774	*Pichia angusta*	119	9.10/26.4	1.51	**8.23**	**
**Cellular metabolism**								
***Protein folding***								
53	Cell division control protein Cdc48	gi|212529968	*Penicillium marneffei*	1376	4.99/90.2	0.00	**1.77**	***
98	ATP-dependent molecular chaperone HSC82	gi|1708315	*Saccharomyces cerevisiae*	118	4.77/80.9	0.00	**6.19**	**
155	Heat shock protein SSC1	gi|310798300	*Glomerella graminicola*	1086	5.82/73.0	1.49	**8.37**	***
180	Heat shock protein SSC1	gi|310798300	*Saccharomyces cerevisiae*	257	5.82/73.0	**5.50**	1.68	***
286	Heat shock protein 60, mitochondrial	gi|123579	*Saccharomyces cerevisiae*	798	5.23/60.8	3.14	**13.92**	***
1437	Ssa1p	gi|144228166	*Saccharomyces cerevisiae*	82	4.82/69.6	1.83	**6.33**	***
***Regulation***								
357	Rab GDP-dissociation inhibitor	gi|729566	*Saccharomyces cerevisiae*	259	5.66/51.2	0.00	**3.97**	***
1065	Protein BMH2	gi|255731125	*Candida tropicalis*	94	4.73/29.4	4.84	**16.10**	**
**Cell process**								
***Replication***								
341	Nucleosome assembly protein	gi|164429322	*Neurospora crassa*	469	4.30/46.3	2.73	**5.62**	*

a Protein names and accession numbers are derived from the NCBI database. ^b^ Mascot scores higher than or equal to 60 are significant (p<0.05). ^c^ Theoretical MW and pI recorded in the NCBI database. ^d^ Average quantity in treated groups. Higher values are in bold type. ^e^ p-values derived from the analysis of variance: * p<0.05, ** p<0.01, *** p<0.001.

#### Proteins down-represented in the presence of *B. cinerea*

Out of the 13 proteins implied in genome expression, only **guanylate kinase** (spot 944) has a function in nucleotide synthesis. The other 12 proteins are implied in protein synthesis pathways. **Methionine synthase** (spots 79 and 80), **homocitrate synthase** (spot 352), **acetohydroxy-acid isomeroreductase** (spot 539), and **NADP-specific glutamate dehydrogenase** (spot 1048) have specific functions in amino acid metabolism. The **translation elongation factor 2** (spot 131), **heat shock protein SSB1** (spots 199, 207 and 213), the **mitochondrial isoform of GTPase elongation factor Tu** (spot 469) and **40S ribosomal protein S0** (spots 696 and 1460) are involved in the translation process.

Six proteins are involved in the carbohydrate and energy metabolisms. **2,3-bisphosphoglycerate-independent phosphoglycerate mutase** (spot 333) and **enolase 1** (spots 441 and 452) are two glycolytic enzymes. The **β-subunit of succinyl-CoA synthetase** (spot 506) belongs to the citric acid cycle and the **β-subunit of the F1F0-ATPase complex** (spot 385) is a constituent of the respiratory chain.

Only one protein, **heat shock protein SSC1** (spot 180) has a function in the cellular regulation metabolism.

#### Proteins over-represented in the presence of *B. cinerea*

Seventeen proteins over-represented in the presence of the pathogen have a function in genome expression. **Adenylosuccinate synthetase** (spots 378 and 399) and **glutamine synthetase** (spot 492) are involved in nucleotide synthesis. **ATP-dependent RNA helicase sub2** (spots 354 and 368) is involved in transcription. The **thiamine pyrophosphate enzyme** (spot 238) is involved in the citric acid cycle and the pentose phosphate pathway (PPP). **NADP-dependent GDH** (spot 336), **S-adenosyl-L-homocysteine hydrolase** (spot 418) and **acetohydroxy-acid isomeroreductase** (spot 1421) are involved in amino acid metabolism. **Glycyl-tRNA synthetase** (spot 135), **heat shock protein SSB1** (spots 222, 225, 300 and 685), **ATP-dependent RNA helicase** (spot 451), **elongation factor 1β** (spot 680) and **translation elongation factor 2** (spot 1433) are implied in translation.

Nineteen proteins are implied in the carbohydrate and energy metabolisms. **Enolase 1** (spots 594 and 683), the **β-subunit of pyruvate dehydrogenase** (spot 623), **fructose-1,6-biphosphate aldolase** (spot 686) and **triosephosphate isomerase** (spot 814) are glycolytic enzymes. **Succinate dehydrogenase** (spot 187) is involved in the citric acid cycle. The **subunit a of vacuolar ATPase V1** (spot 194), **subunit 1 of the cytochrome Bc1 complex** (spot 375), the **β-chain of ATP synthase** (spot 382), **inorganic pyrophosphatase** (spot 609), the **β-subunit of F-ATPase** (spot 736), **subunit VI of cytochrome c oxidase** (spot 1412) and **subunit b of the stator stalk of F1F0 ATP synthase** (spot 1444) are proteins involved in the oxidative phosphorylation pathway. **6-phosphogluconate dehydrogenase** (spots 351 and 390), **transketolase** (spots 120 and 121) and **transaldolase** (spot 631) are implied in the PPP. **Mannose-1-phosphate guanyltransferase** (spot 498) and **L-xylulose reductase** (spot 724), two enzymes of the pentose and glucuronate interconversion pathways, were also influenced.

Eight proteins have a role in various cellular processes. Cell division control protein Cdc48 (spot 53), ATP-dependent molecular chaperone HSC82 (spot 98), heat shock protein SSC1 (spot 155), the mitochondrial isoform of heat shock protein 60 (spot 286) and heat shock protein SSA1p (spot 1437) are involved in protein folding. Rab GDP-dissociation inhibitor (spot 357) and protein BMH2 (spot 1065) have a function in metabolic pathway regulation. Nucleosome assembly protein (spot 341) is a protein involved in the mitosis process.

### Proteome changes during the stationary phase

Table 3 shows proteins influenced by the absence or presence of *B. cinerea* during the stationary phase. Twenty proteins were down-represented, while 40 were over-represented in the presence of the pathogen.

**Table 3:** Identification of proteins exhibiting different expression levels according to the absence (Kh6-2) or the presence (Kh6BB-2) of *B. cinerea* in the stationary phase.

Spot no.	Protein name^a^	Accession no.^a^	Organism	Mascot score^b^	Theoretical pI/MW^c^	Spot quantity (ppm)^d^		p^e^
						Kh6-1	Kh6B-1	
**Genome expression**								
***Nucleotide metabolism***								
471	Glutamine synthetase	gi|149239324	*Lodderomyces elongisporus*	114	5.81/41.7	1.59	3.69	*
850	Orotate phosphoribosyltransferase	gi|3024492	*Saccharomyces cerevisiae*	435	5.13/24.8	0.63	1.28	*
***Amino acid metabolism***								
343	NADP-specific glutamate dehydrogenase	gi|225560463	*Ajellomyces capsulatus*	107	5.5/49.6	2.40	5.58	*
530	Acetohydroxy-acid isomeroreductase	gi|957238	*Saccharomyces cerevisiae*	254	9.06/44.4	5.77	11.51	*
***Translation***								
662	40S ribosomal protein S0	gi|119490951	*Neosartorya fischeri*	282	4.47/28.0	6.77	12.62	**
973	Eukaryotic translation initiation factor 5A	gi|320583352	*Pichia angusta*	102	7.23/75.3	4.55	7.48	*
**Carbohydrate metabolism**								
***Glycolysis***								
363	Phosphoglycerate kinase	gi|57157302	*Candida boidinii*	94	6.16/44.3	2.94	6.67	*
534	Fructose 1,6-bisphosphate aldolase	gi|254565205	*Pichia pastoris*	86	6.02/39.7	4.58	7.16	*
765	Phosphoglycerate mutase	gi|254571899	*Thermobifida fusca*		5.85/27.7	3.61	6.23	*
814	Enolase 1	gi|238850305	*Clavispora lusitaniae*	162	5.55/47.1	3.46	6.03	*
1177	Glyceraldehyde-3-phosphate dehydrogenase, isozyme 3	gi|2494641	*Pichia pastoris*	51	6.24/35.6	8.67	3.39	***
1344	Pyruvate kinase	gi|151945424	*Saccharomyces cerevisiae*	83	6.43/55.2	0.44	0.88	*
1350	Fructose 1,6-bisphosphate aldolase	gi|254565205	*Pichia pastoris*	76	6.02/39.7	21.87	11.84	*
1377	Fructose 1,6-bisphosphate aldolase	gi|254565205	*Pichia pastoris*	76	6.02/39.7	6.24	3.85	***
***Citric acid cycle***								
102	Aconitase 1	gi|6323335	*Saccharomyces cerevisiae*	1300	8.17/85.2	1.77	3.80	*
***Alcoholic fermentation***								
241	Pyruvate decarboxylase	gi|6323073	*Saccharomyces cerevisiae*	110	5.45/53.0	7.62	0.00	*
247	Pyruvate decarboxylase	gi|6323073	*Saccharomyces cerevisiae*	79	5.45/53.0	2.11	0.88	*
261	Pyruvate decarboxylase	gi|111607053	*Pichia anomala*	93	5.45/53.0	1.45	2.34	*
264	Pyruvate decarboxylase	gi|111607053	*Pichia anomala*	92	5.45/53.0	0.00	1.40	***
322	Pyruvate decarboxylase	gi|111607053	*Pichia anomala*	53	5.45/53.0	2.60	0.91	**
Energetic metabolism								
***Oxidative phosphorylation***								
1135	Mitochondrial F-ATPase beta subunit	gi|91178130	*Pichia anomala*	70	4.78/36.2	3.52	1.93	*
**Cellular metabolism**								
***Regulation***								
939	Thioredoxin peroxidase	gi|6323613	*Saccharomyces cerevisiae*	102	4.87/21.6	1.28	0.00	**
1079	Protein BMH2	gi|255731125	*Candida tropicalis*	93	4.73/29.4	3.65	0.84	***

a Protein names and accession numbers are derived from the NCBI database. ^b^ Mascot scores higher than or equal to 60 are significant (p<0.05). ^c^ Theoretical MW and pI recorded in the NCBI database. ^d^ Average quantity in treated groups. Higher values are in bold type. ^e^ p-values derived from the analysis of variance: * p<0.05, ** p<0.01, *** p<0.001.

Proteins down-represented in the presence of *B. cinerea*. Seven proteins are implied in the carbohydrate and energy metabolisms. Glyceraldehyde-3-phosphate dehydrogenase (spot 1177) and fructose-1,6-biphosphate aldolase (spots 1350 and 1377) are glycolytic enzymes. Pyruvate decarboxylase (spots 241, 247 and 322) is implied in alcoholic fermentation. Mitochondrial F-ATPase β (spot 1135) is a constituent of the respiratory chain.

Moreover, **thioredoxin peroxidase** (spot 939) and **protein** **BMH2** (spot 1079) are involved in regulation processes.

#### Proteins over-represented in the presence of *B. cinerea*

Six proteins over-represented in the presence of the pathogen are involved in genome expression. **glutamine synthetase** (spot 471) and **orotate phosphoribosyltransferase** (spot 850) have a role in nucleotide synthesis. **NADP-specific glutamate dehydrogenase** (spot 343) and **acetohydroxy-acid isomeroreductase** (spot 530) are involved in amino acid metabolism. **40S ribosomal protein S0** (spot 662) and **eukaryotic translation initiation factor 5A** (spot 973) are translational enzymes.

Eight proteins have a function in carbohydrate metabolism. **Phosphoglycerate kinase** (spot 363), **fructose-1,6-biphosphate aldolase** (spot 534), **phosphoglycerate mutase** (spot 765), **enolase 1** (spot 814) and **pyruvate kinase** (spot 1344) are glycolytic enzymes. **Aconitase 1** (spot 102) is involved in the citric acid cycle and **pyruvate decarboxylase** (spots 261 and 264) is implied in alcoholic fermentation.

## Discussion

The objective of the present study was to identify metabolic pathways of *P. anomala* influenced by the presence of *B. cinerea*, under conditions close to natural infection conditions. This allowed us to correlate proteome expression with the biocontrol process and propose molecular links between both datasets. Exponential and stationary phase proteomic profiles differed, suggesting different physiological states of the yeast. The proteome of *P. anomala* inoculated alone on apple wounds was compared to the proteome of *P. anomala* co-inoculated with *B. cinerea,* first in the exponential growth phase and second in the stationary growth phase. The large number of proteins influenced by the presence of *B. cinerea* in the exponential compared to the stationary phase seemed to indicate that the early response of *P. anomala* to the introduction of the pathogen involves large metabolic modifications.

### The pentose phosphate pathway: a possible early answer to the introduction of *B. cinerea*

In the early stages after inoculation, *i.e.* in the exponential growth phase, our results may indicate that *P. anomala* modified its energetic metabolism in order to respond to its needs.

In the absence of *B. cinerea*, the over-representation of two isoforms of **enolase 1** (spots 441 and 452) and **2,3-bisphosphoglycerate-independent phosphoglycerate mutase** (spot 333) suggests that the glycolysis pathway is activated during the growth and wound colonization. Indeed, glyceraldehyde-3-phosphate dehydrogenase and other glycolytic enzymes were the most abundant proteins isolated from *Saccharomyces cerevisiae* grown on a glucose medium in the exponential phase [29]. Apples are composed of 12.6% carbohydrates, mainly in the form of glucose-derived molecules such as fructose (a glucose isomer), sorbitol and pectin (a heteropolysaccharide of galacturonic acid, arabinose, galactose, rhamnose and xylose [30]) that could enter the glycolytic pathway to provide *P. anomala* with the energy required for its growth. Likewise; the higher proportion of proteins involved in the protein synthesis process in the absence of *B. cinerea* should be the consequence of the high rate of protein synthesis that occurs in the exponential phase. Indeed, the highest incorporation rate of 35S-amino acids in a culture of *S. cerevisiae* on YPD medium was observed during the exponential phase [31].

**Figure 2:**
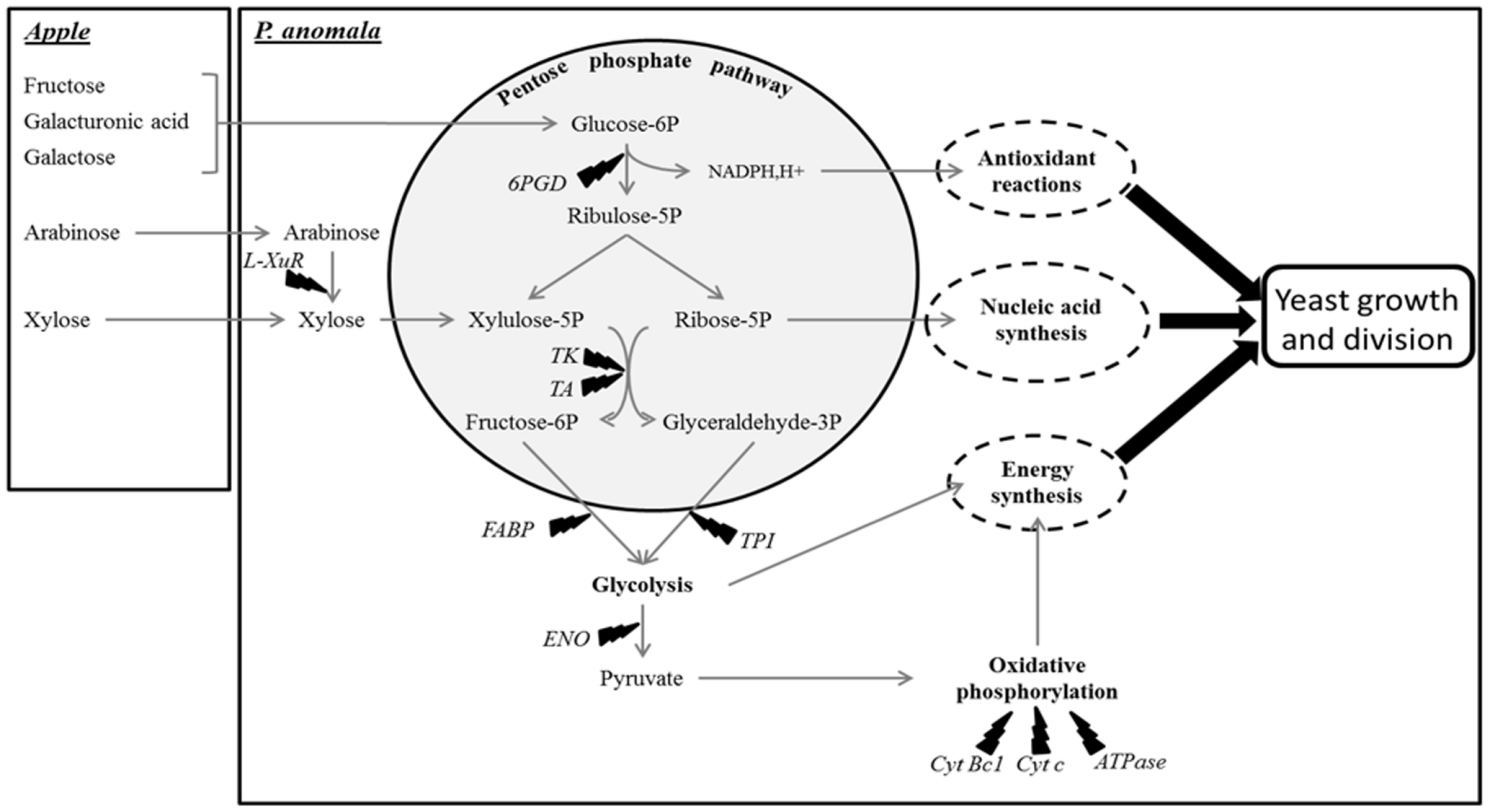
Diagram showing the implication of the pentose phosphate pathway in the mode of action of the antagonist *P. anomala.*

The proteomic map of *P. anomala* in the presence of *B. cinerea* during the exponential phase showed that proteins involved in the carbohydrate and energy metabolisms, genome expression and cellular metabolism were highly represented, suggesting a high metabolic activity. This high activity may be related to the capacity of *P. anomala* to exhibit similar growth time-courses in the absence or presence of *B. cinerea* (Figure 1) [27]. In the presence of the pathogen, proteins involved in the PPP are over-represented and may allow *P. anomala* to efficiently use apple nutrients and support growth. Arabinose, an apple constituent, can be converted into xylose by **L-xylulose reductase** (spot 724, Table 2 and Fig. 2), an enzyme of the pentose and glucuronate interconversion pathway [32] that is over-represented in the presence of *B. cinerea*. PPP has been demonstrated as the main route for xylose catabolism in yeast [33]. The newly produced amount of xylose from arabinose or xylose derived from apple could then enter the PPP (Fig. 2) under the xylulose-5P form and be converted into fructose-6P and glyceraldehyde-3P, two glycolysis intermediates, *via* the action of **transketolase** (spots 120 and 121) and **transaldolase** (spot 631, Fig. 2), two enzymes over-represented in the presence of *B. cinerea*. The over-representation of five glycolysis proteins, particularly **triosephosphate isomerase** (spot 814) and **fructose-1,6-biphosphate aldolase** (spot 686) suggests the entry of fructose-6P and glyceraldehyde-3P into glycolysis and the production of high amount of pyruvate. Then pyruvate may be metabolised to supply substrates for oxidative phosphorylation inside mitochondria as suggested by the over-representation of seven proteins involved in that pathway (Table 2 and Fig. 2).

Moreover, fructose, sorbitol, galactose and galacturonic acid, other apple constituents could be converted into glucose-6P. The over-representation of **6-phosphogluconate dehydrogenase** (spots 351 and 390), the first enzyme of the PPP which catalyses the irreversible decarboxylation of 6-phosphogluconate into ribulose-5-phosphate, suggests that the newly-formed glucose-6P could enter the PPP to form ribulose-5P together with synthesis of NADPH,H+ (Fig. 2), a powerful reducing-oxidative agent involved in protection against the toxicity of reactive oxygen species and in glutathione regeneration [34], [35]. The newly synthesised NADPH,H+ may probably be used during oxidative phosphorylation in order to protect the yeast against reactive oxygen species (Fig. 2). Indeed, PPP plays an important role in the oxidative stress response in *Saccharomyces cerevisiae* [36].

These results are linked to the over-representation of 17 proteins involved in nucleotide metabolism, transcription, amino acid metabolism and translation. *Via* the PPP, newly-synthesised ribulose-5P may be converted into ribose-5P, the precursor of nucleic acids (Fig. 2), in order to respond to its high metabolic activity in the presence of *B. cinerea*. Moreover, the capacity of *P. anomala* to exhibit similar growth time-courses in the absence or presence of *B. cinerea* (Fig. 1) and the over-representation of proteins involved in cell division, **cell division control protein Cdc48** (spot 53) implicated in protein processing in the endoplasmic reticulum, **ATP-dependent molecular chaperone HSC82** (spot 98), a constitutively expressed protein involved in the cellular cycle [37], and **Nucleosome assembly protein** (spot 341), a protein involved in mitosis [38], suggest that *P. anomala* could mainly use ribose-5-P for its multiplication and growth.

Thus, in the presence of *B. cinerea*, our results suggest that the PPP may supply the yeast with an efficient consumption of apple nutrients and consequently an adaptation of its metabolism to meet its immediate needs. Then, we could hypothesize that *P. anomala* may be an efficient coloniser of the wound and a nutrient competitor for *B. cinerea*. Actually, competition for space of specific infection sites is admitted as a mode of action of various biocontrol agents [7]–[12]. Our results may link the PPP with the protective effect of *P. anomala* against *B. cinerea* on apple. However, additional experiments have to be done in order to confirm this hypothesis. For example, the gene coding for **6-phosphogluconate dehydrogenase** could be disrupted in order to inhibit the PPP and observe its influence on the protection level.

### The alcoholic fermentation: the energy metabolism in the later stages

In the later stages after inoculation, *i.e.* in the stationary growth phase, the proteome of *P. anomala* in the absence or the presence of *B. cinerea* seems to present no differences in the carbohydrate and energy metabolism. Proteins implied in glycolysis (spots 363, 534, 765, 814, 1177, 1344, 1350 and 1377) and alcoholic fermentation (**pyruvate decarboxylase**, spots 241, 247, 261, 264 and 322) are expressed. Alcoholic fermentation was further identified as a metabolic pathway providing energy in the post-diauxic phase that precedes the stationary phase. On the contrary, when yeast was grown on a rich medium for a long time, energy was provided by oxidative respiration in the stationary phase [39]. According to our growth time-course (Figure 1), 24h after inoculation, *P. anomala* presented stagnation in its population suggesting that yeast entered the stationary phase [27]. Then *P. anomala* should use the oxidative respiration and not the alcoholic fermentation. However, another study demonstrates that *P. anomala*, when inoculated on a glucose-rich medium, used alcoholic fermentation as a way to provide energy in response to oxygen limitation [40]. In our model, inoculated apples were enclosed in plastic boxes in order to maintain a high relative humidity. Moreover, wounded apple sites represent media that are rich in glucose-derived molecules. During the first growth steps, the two microorganisms may possibly consume a large part of the available oxygen, or oxygen proportion may have been modified by microorganism carbon dioxide release or apple ethylene release. In these conditions, oxygen limitation may induce fermentative metabolism in *P. anomala* [40].

These last results could be a new way to explore in biocontrol. Indeed; in enclosed conditions, as in our experiment, yeast could use alcoholic fermentation and may produce volatile compounds like ethanol or ethyl acetate recently described as possible antimicrobial and antifungal compounds [41]. Then we could hypothesize that this volatile compound production could then contribute to the *B. cinerea* biocontrol by *P. anomala*. Finally, monitoring oxygen availability in storage rooms could improve the efficiency of biocontrol by *P. anomala*. However, we cannot rule out the implication of competition for space and nutrients in the inhibitory effect against the pathogen in the later stages. In the stationary phase, yeast was totally established on the wound site, leaving no space or nutrients for *B. cinerea* growth. Thus, *P. anomala* could maintain its inhibitory effect on the pathogen without necessarily inducing any specific metabolic pathway involved in the biocontrol modes of action, as in the exponential phase. However, additional experiments have to be done in order to confirm this hypothesis.

### Observation of a higher protein synthesis induced by the introduction of the pathogen in the later stages

In the later stages after the co-inoculation, proteins implied in genome expression seem to be over-represented: **glutamine synthetase** (spot 471), which catalyses the fixation of a second amine group on glutamate to synthetise glutamine (ter Schure et al., 2000), **orotate phosphoribosyltransferase** (spot 850), which catalyses orotidine monophosphate formation during pyrimidine metabolism [42], **NADP-dependent glutamate dehydrogenase** (spot 343), involved in the conversion of α-ketoglutarate into glutamate [34], [43], **acetohydroxy-acid isomeroreductase** (spot 530), involved in valine, leucine and isoleucine synthesis [44], [45], **40S ribosomal protein S0** (spot 662), which is required for the assembly and/or stability of the 40S ribosomal subunit [46] and **eukaryotic translation initiation factor 5A** (spot 973), which plays a role in the formation of the first peptide bond [47]. This would suggest a higher protein synthesis rate than in the absence of the pathogen. Introducing the pathogen induced metabolic modifications such as the implementation of new metabolic pathways during the exponential phase. Here, the over-representation of proteins implied in genome expression could be the first signs of a new metabolic pathway or alternatively, in the presence of the pathogen, yeast may have maintained a high level of alcoholic fermentation activity implying high corresponding enzyme synthesis levels.

Finally, this study aimed to identify *P. anomala* metabolic pathways that are differentially expressed after the introduction of *B. cinerea* on apple. In the early stages of the co-inoculation *P. anomala* seems to set up the pentose phosphate pathway in order to supply higher amounts of energy and nucleic acid and support its high metabolic activity. This result suggests that PPP supports the efficient apple wound colonization. In the later stages, *P. anomala* seems to use alcoholic fermentation which is associated with an increased protein synthesis potential.

Nevertheless, this study confirms the complexity of the interaction between *B. cinerea* and *P. anomala*. Different mechanisms are influenced by growth stages and experimental conditions. However, these results suggested new targets in the study of the yeast mode of action against the pathogen in apple. Different pathways could be investigated in order to improve our knowledge of the biocontrol agent *P. anomala*.

## References

[1] Abano EE, Sam-Amoah LK (2012) Application of antagonistic microorganisms for the control of postharvest decays in fruits and vegetables. Int J Adv Biol Res 2: 1–8.

[2] Janisiewicz WJ, Korsten L (2002) Biological control of postharvest diseases of fruits. Annu Rev Phytopathol 40: 411–441. 12147766 10.1146/annurev.phyto.40.120401.130158

[3] Jijakli MH (2011) *Pichia anomala* in biocontrol for apples: 20 years of fundamental research and practical applications. J Gen Mol Microbiol 99: 93–105. 10.1007/s10482-010-9541-2 21222032

[4] Fravel DR (2005) Commercialization and implementation of biocontrol. Annu Rev Phytopathol 43: 337–359. 16078888 10.1146/annurev.phyto.43.032904.092924

[5] Droby S, Wisniewski M, Macarisin D, Wilson C (2009) Twenty years of postharvest biocontrol research: Is it time for a new paradigm? Postharvest Biol Tec 52: 137–145.

[6] Castoria R, De Curtis F, Lima G, Caputo L, Pacifico S, et al (2001) *Aureobasidium pullulans* (LS-30) an antagonist of postharvest pathogens of fruits: study on its modes of action. Postharvest Biol Tec 22: 7–17.

[7] Piano S, Neyrotti V, Migheli Q, Gullino ML (1997) Biocontrol capability of *Metschnikowia pulcherrima* against *Botrytis* postharvest rot of apple. Postharvest Biol Tec 11: 131–140.

[8] Spadaro D, Vola R, Piano S, Gullino ML (2002) Mechanisms of action and efficacy of four isolates of the yeast *Metschnikowia pulcherrima* active against postharvest pathogens on apples. Postharvest Biol Tec 24: 123–134.

[9] Filonow AB (1998) Role of competition for sugars by yeasts in the biocontrol of gray mold of apple. Biocontrol Sci Techn 8: 243–256.

[10] Droby S, Chalutz E, Wilson CL, Wisniewski M (1989) Characterization of the biocontrol activity of *Debaryomyces hansenii* in the control of *penicillium digitatum* on grapefruit. Can J Microbiol 35: 794–800.

[11] Wisniewski M, Wilson C, Hershberger W (1989) Characterization of inhibition of *Rhizopus stolonifer* germination and growth by *Enterobacter cloacae* . Can J Botanic 67: 2317–2323.

[12] Andrews JH, Harris RF, Spear RN, Lau GW, Nordheim EV (1994) Morphogenesis and adhesion of *Aureobasidium pullulans* . Can J Microbiol 40: 6–17.

[13] Jijakli MH, Lepoivre P (1998) Characterization of an Exo-β-1,3-Glucanase produced by *Pichia anomala* Strain K, antagonist of *Botrytis cinerea* on apples. Phytopathol 88: 335–343. 10.1094/PHYTO.1998.88.4.335 18944957

[14] Wisniewski M, Biles C, Droby S, McLaughlin R, Wilson C, et al (1991) Mode of action of the postharvest biocontrol yeast, *Pichia guilliermondii*.1. characterization of attachment to *Botrytis cinerea* . Physiol Mol Plant Pathol 39: 245–258.

[15] Spadaro D, Gullino ML (2004) State of the art and future prospects of the biological control of postharvest fruit diseases. Int J Food Microbiol 91: 185–194. 14996462 10.1016/S0168-1605(03)00380-5

[16] Bajji M, Jijakli MH (2009) cDNA-AFLP analysis of *Candida oleophila* (strain O) genes differentially expressed during the biocontrol *of Botrytis cinerea* on harvested apples. IOBC/wprs Bulletin 43: 327–330.

[17] Jijakli MH, Lepoivre P, Tossut P, Thonart P (1993) Biological control of *Botrytis cinerea* and *Penicillium* on postharvest apples by two antagonistic yeasts. Med Fac Landbouwwet, University of Gent 58: 1349–1358.

[18] Grevesse C, Lepoivre P, Jijakli MH (2003) Characterization of the Exoglucanase-encoding gene PaEXG2 and study of its role in the biocontrol activity of *Pichia anomala* Strain K. . Phytopathol 93: 1145–1152. 10.1094/PHYTO.2003.93.9.1145 18944099

[19] Jijakli MH, Lepoivre P, Grevesse C (1999) Yeast species for biocontrol of apples postharvest diseases: an encouraging case of study for practical use. Biotechnol app biocontrol plant path 31–49.

[20] Massart S, Jijakli MH (2006) Identification of differentially expressed genes by cDNA-amplified fragment length polymorphism in the biocontrol agent *Pichia anomala* (Strain Kh5). Phytopathol 96: 80–86. 10.1094/PHYTO-96-0080 18944207

[21] Friel D, Pessoa NMG, Vandenbol M, Jijakli MH (2007) Separate and combined disruptions of two exo-beta-1,3-glucanase genes decrease the efficiency of *Pichia anomala* (strain K) biocontrol against *Botrytis cinerea* on apple. Mol Plant-Microbe In 20: 371–379. 10.1094/MPMI-20-4-0371 17427807

[22] Jijakli MH, Lepoivre P (1993) Biological control of postharvest *Botrytis cinerea* and *Penicillium* on apples. IOBC/WRPS Bulletin: Biological control of foliar and Post-harvest Diseases 16: 106–110.

[23] Gorg A, Weiss W, Dunn MJ (2005) Current two dimensional electrophoresis technology for proteomics. Proteomics 5: 826–827. 10.1002/pmic.200401031 15543535

[24] Marra R, Ambrosino P, Carbone V, Vinale F, Woo SL, et al (2006) Study of the three-way interaction between *Trichoderma atroviride*, plant and fungal pathogens by using a proteomic approach. Curr Genet 50: 307–321. 17008992 10.1007/s00294-006-0091-0

[25] Grinyer J, McKay M, Nevalainen H, Herbert BR (2004) Fungal proteomics: initial mapping of biological control strain *Trichoderma harzianum* . Curr Genet 45: 163–169. 14685766 10.1007/s00294-003-0474-4

[26] Grinyer J, Hunt S, McKay M, Herbert BR, Nevalainen H (2005) Proteomic response of the biological control fungus *Trichoderma atroviride* to growth on the cell walls of *Rhizoctonia solani* . Curr Genet 47: 381–388. 15856359 10.1007/s00294-005-0575-3

[27] Kwasiborski A, Bajji M, Delaplace P, Lepoivre P, Jijakli MH (2012) Biocontrol proteomics: Development of an in situ method for a proteomic study of inhibition mechanisms of *Pichia anomala* against *Botrytis cinerea* on apple. Biocontrol 57: 837–848.

[28] Delaplace P, Wall FVd, Dierick J-F, Cordewener JHG, Fauconnier M-L, et al (2006) Potato tuber proteomics: Comparison of two complementary extraction methods designed for 2-DE of acidic proteins. Proteomics 6: 6494–6497. 17096317 10.1002/pmic.200600493

[29] Norbeck J, Blomberg A (1997) Two-dimensional electrophoretic separation of yeast proteins using a non-linear wide range (pH 3–10) immobilized pH gradient in the first dimension; reproducibility and evidence for isoelectric focusing of alkaline (pI 7) proteins. Yeast 13: 1519–1534. 9509572 10.1002/(SICI)1097-0061(199712)13:16<1519::AID-YEA211>3.0.CO;2-U

[30] Barrett AJ, Northcote DH (1965) Apple fruit pectic substances. Biochem J 94: 617–627. 14340052 10.1042/bj0940617 PMC1206596

[31] Fuge EK, Braun EL, Werner-Washburne M (1994) Protein synthesis in long-term stationary-phase cultures of *Saccharomyces cerevisiae* . J Bacteriol 176: 5802–5813. 8083172 10.1128/jb.176.18.5802-5813.1994 PMC196785

[32] Nair N, Zhao H (2007) Biochemical characterization of an L-Xylulose reductase from *Neurospora crassa* . App Env Microbiol 73: 2001–2004. 10.1128/AEM.02515-06 PMC1828828 17261518

[33] Matsushika A, Goshima T, Fujii T, Inoue H, Sawayama S, et al (2012) Characterization of non-oxidative transaldolase and transketolase enzymes in the pentose phosphate pathway with regard to xylose utilization by recombinant *Saccharomyces cerevisiae* . Enzyme Microb Tech 51: 16–25. 10.1016/j.enzmictec.2012.03.008 22579386

[34] Bogonez E, Satrustegui J, Machado A (1985) Regulation by ammonium of glutamate dehydrogenase (NADP+) from *Saccharomyces cerevisiae* . J Gener Microbiol 131: 1425–1432. 10.1099/00221287-131-6-1425 2995545

[35] Ying W (2007) NAD+/NADH and NADP+/NADPH in cellular functions and cell death: regulation and biological consequences. Antioxid Redox Sign 10: 179–206. 10.1089/ars.2007.1672 18020963

[36] Andrew EJ, Merchan S, Lawless C, Banks AP, Wilkinson DJ, et al.. (2013) Pentose phosphate pathway function affects tolerance to the g-quadruplex binder TMPyP4. Plos One 8. 10.1371/journal.pone.0066242 PMC3680382 23776642

[37] Borkovich KA, Farrelly FW, Finkelstein DB, Taulien J, Lindquist S (1989) HSP82 is an essential protein that is required in higher concentrations for growth of cells at higher temperatures. Mol Cell Biol 9: 3919–3930. 2674684 10.1128/mcb.9.9.3919-3930.1989 PMC362454

[38] Donoso I, Munoz-Centeno MC, Sanchez-Duran MA, Flores A, Daga RR, et al (2005) Mpg1, a fission yeast protein required for proper septum structure, is involved in cell cycle progression through cell-size checkpoint. Mol Genet Genomics 274: 155–167. 16049679 10.1007/s00438-005-0005-8

[39] Zakrajsek T, Raspor P, Jamnik P (2011) *Saccharomyces cerevisiae* in the stationary phase as a model organism - characterization at cellular and proteome level. J Proteomics 74: 2837–2845. 21782986 10.1016/j.jprot.2011.06.026

[40] Fredlund E, Blank LM, Schnurer J, Sauer U, Passoth V (2004) Oxygen- and glucose-dependent regulation of central carbon metabolism in *Pichia anomala* . Appl Environ Microb 70: 5905–5911. 10.1128/AEM.70.10.5905-5911.2004 PMC522099 15466531

[41] Fredlund E, Druvefors UA, Olstorpe MN, Passoth V, Schnurer J (2004) Influence of ethyl acetate production and ploidy on the anti-mould activity of *Pichia anomala* . Fems Microbiol Lett 238: 133–137. 15336413 10.1016/j.femsle.2004.07.027

[42] Victor J, Greenberg LB, Sloan DL (1979) Studies of the kinetic mechanism of orotate phosphoribosyltransferase from yeast. J Biol Chem 254: 2647–2655. 218950

[43] ter Schure EG, van Riel NAW, Verrips CT (2000) The role of ammonia metabolism in nitrogen catabolite repression in *Saccharomyces cerevisiae* . Fems Microbiol Rev 24: 67–83. 10640599 10.1111/j.1574-6976.2000.tb00533.x

[44] Primerano DA, Burns RO (1983) Role of acetohydroxy acid isomeroreductase in biosynthesis of pantothenic acid in *Salmonella typhimurium* . J Bacteriol 153: 259–269. 6401279 10.1128/jb.153.1.259-269.1983 PMC217364

[45] Dumas R, Biou V, Halgand F, Douce R, Duggleby RG (2001) Enzymology, structure, and dynamics of acetohydroxy acid isomeroreductase. Accounts Chem Res 34: 399–408. 10.1021/ar000082w 11352718

[46] Demianova M, Formosa TG, Ellis SR (1996) Yeast proteins related to the p40/laminin receptor precursor are essential components of the 40 S ribosomal subunit. J Biol Chem 271: 11383–11391. 8626693 10.1074/jbc.271.19.11383

[47] Zanelli CF, Maragno ALC, Gregio APB, Komili S, Pandolfi JR, et al (2006) eIF5A binds to translational machinery components and affects translation in yeast. Biochem. Bioph. Res. Comm 348: 1358–1366. 10.1016/j.bbrc.2006.07.195 16914118

